# Expression and Clinical Significance of YAP, TAZ, and AREG in Hepatocellular Carcinoma

**DOI:** 10.1155/2014/261365

**Published:** 2014-04-22

**Authors:** Su-xia Han, E. Bai, Gui-hua Jin, Chen-chen He, Xi-jing Guo, Li-juan Wang, Meng Li, Xia Ying, Qing Zhu

**Affiliations:** ^1^Department of Medical Oncology, The First Affiliated Hospital of Xi'an Jiaotong University Medical College, Xi'an, Shaanxi 710061, China; ^2^Center of Maternal and Child Health, The First Affiliated Hospital of Xi'an Jiaotong University Medical College, Xi'an, Shaanxi 710061, China

## Abstract

*Purpose*. Yes-associated protein (YAP) and PDZ-binding motif (TAZ) are two important effectors of Hippo pathway controlling the balance of organ size and carcinogenesis. Amphiregulin (AREG) is a member of the epidermal growth factor family, a direct target gene of YAP and TAZ. The role of these proteins in hepatocellular carcinoma (HCC) is unclear. *Methods*. The expression of YAP, TAZ, and AREG in HCC was analyzed by immunohistochemical staining. The level of secreted serum AREG was also assayed by enzyme-linked immunosorbent (ELISA) assay. *Results*. YAP, TAZ, and AREG were expressed in 69.2% (27/39), 66.7% (26/39), and 61.5% (24/39) of HCC patients. The expression of YAP was significantly correlated with Edmondson stage (*P* > 0.05), serum AFP level (*P* > 0.05), and HCC prognosis (*P* > 0.05). AREG expression was also significantly correlated with Edmondson stage (*P* > 0.05) and serum AFP level (*P* > 0.05). In addition, the expression of serum AREG was higher than serum AFP in HCC patients. Further multivariate analysis showed that YAP expression was an independent prognostic factor that significantly affected the overall survival of HCC patients. *Conclusions*. YAP maybe an independent prognostic indicator for HCC patients and serum AREG may be a serological biomarker of HCC.

## 1. Introduction

Hepatocellular carcinoma (HCC) is the sixth most common cancer worldwide and is the second leading cause of cancer-related death in China. HCC usually develops in patients with chronic inflammatory liver disease such as viral infection and/or exposure to chemical carcinogens [1]. Although much progress has been made using multiple therapeutic approaches to HCC, the majority of patients are diagnosed at an advanced stage at which point treatments are ineffective. Early detection and diagnosis and prompt treatment might improve the life quality and survival of HCC patients.

Yes-associated protein (YAP) and transcriptional coactivator with PDZ-binding motif (TAZ) were identified as Yki homologs in mammals [2]. These Yki homologs are phosphorylated and inhibited by the Hippo pathway through cytoplasmic retention [3]. TAZ was initially identified through its ability to interact with 14-3-3 proteins and is also called WWTR1 (WW domain-containing transcription regulator 1). TAZ contains a conserved WW domain capable of interacting with a PPXY motif, a coiled-coil region implicated in protein-protein interaction, and a COOH terminal motif capable of interacting with the PDZ domain [4]. TAZ and YAP are two important effectors of the Hippo pathway which controls the balance of organ size and carcinogenesis. The inactivation of Hippo pathway could lead to excessive cell proliferation, inhibition of apoptosis resulting in carcinogenesis. It was shown that the expressions of YAP and TAZ were elevated in multiple types of human cancers, such as breast cancer and lung cancer. In human HCC, some studies revealed that YAP functions as a tumor suppressor [5] and others demonstrated that YAP was an oncogene [6, 7]. Amphiregulin (AREG) is a member of the epidermal growth factor family that combines with the epidermal growth factor receptor (EGFR) and activates the downstream of signalling pathway. AREG, a secreted protein, was also observed to promote cell proliferation and inhibit cell apoptosis in a variety of tumors and could play an important role in extracellular matrix environment or blood circulation [8]. Recently, AREG was shown to be a direct target gene of YAP and TAZ [9, 10]. However, little is known about the expression role of YAP, TAZ, and AREG in HCC and the influence on the prognosis of HCC patients.

In this study, we analyzed the expression and correlation of YAP, TAZ, and AREG in HCC and adjacent tissues by immunohistochemical staining. The level of secreted AREG in sera was also assayed by enzyme-linked immunosorbent assay. The relationship between YAP, TAZ, and AREG expression, serum AREG, and clinicopathological features was also investigated to understand their role in HCC. In addition, we investigated the prognostic value of YAP, TAZ, AREG, and serum AREG, in a retrospective cohort study with a three-year follow-up, to determine the independent predictive role of YAP, TAZ, and AREG in HCC patients.

## 2. Materials and Methods

### 2.1. Patients and Tissue/Sera

Tumor samples and their paired adjacent nontumor tissues were collected, and the pre- and postoperative sera of 39 HCC patients were obtained from the First Affiliated Hospital of Xi'an Jiaotong University from June 2010 to May 2012. These HCC patients included 35 males and 4 females, and the median age of them was 52 years old (ranged from 13 to 74 years old). Sera samples (*n* = 98) were collected from patients in the First Affiliated Hospital, including 50 HCC patients (all HCC patients were histologically confirmed), 31 patients with benign liver diseases, and 17 normal controls. The study was approved by the Institutional Review Board of Xi'an Jiaotong University and collaborating institutions. All patients signed informed consent forms. All patients had not received preoperative chemotherapy, radiotherapy, and embolization and were classified according to the criteria of American Joint Committee on Cancer Staging Manual, Seventh Edition (2010) for HCC. The patients were grouped according to age, sex, tumor size, stage, Edmondson-Steiner classification, hepatitis, presence of portal venous invasion, number of tumor nodules, and serum alpha-fetoprotein (AFP) levels. These HCC patients were followed up over a two-year period. Overall survival of HCC patients was calculated as the time from diagnosis to the date of death. Patients who were alive were treated as censored for overall survival analysis.

### 2.2. Immunohistochemical Staining

Paraffin-embedded tissue sections were collected. After deparaffinized with xylene and dehydrated with ethanol, the tissue sections were subject to microwave antigen retrieval procedure in sodium citrate buffer for 10 min followed by endogenous peroxidase blocking. Sections were then separately incubated with the primary antibodies directed against YAP, TAZ, and AREG at 4°C overnight, detected using biotinylated secondary antibodies (Zhongshan Golden bridge Biotechnology Ltd. Co., China) according to the manufacturer's recommendations. The staining of the sections was performed using the HRP-streptavidin conjugates for YAP, TAZ, and AREG. The sections were visualized with diaminobenzidine and counterstained with hematoxylin. The primary rabbit monoclonal anti-YAP (number 2060-1) and polyclonal anti-TAZ antibody (number T3467) were purchased from Epitomics Incorporated (USA). The primary mouse monoclonal anti-AREG antibody (sc-74501) was purchased from Santa Cruz Biotechnology (USA).

All sections were scored independently by three experienced pathologists. The expression levels were semiquantitatively analyzed by an immunohistochemical score combined with the percentage of hepatic cells showing specific immunoreactivity. Staining intensity was graded as 0, none; 1, weak; 2, moderate; and 3, strong. The percentage of positive stained cells was graded as 0, <5%; 1, 6–25%; 2, 26–50%; 3, 51–75%; and 4, >75%. The total score was calculated by multiplying the staining intensity and the percentage of positive stained cells. Sections with a total score of >4 were defined as exhibiting positive staining for the above proteins.

### 2.3. Enzyme-Linked Immunosorbent Assay for Serum AREG

Serum AREG concentration was measured using commercially available AREG ELISA kits (number KA1695, Abnova Corporation, Taiwan) following the manufacturer's instructions in 96-well flexible microtiter plates. The wells were washed and biotinylated anti-AREG antibody was added. After washing away unbound biotinylated antibody, HRP-conjugated streptavidin was pipetted to the wells. These wells were washed again, a TMB substrate solution was used as the detecting agent. The OD of each well was read at 450 nm.

### 2.4. Statistical Analysis

Statistical analyses were done with SPSS 16.0 (USA). The expressions of the proteins in tumor tissues and their adjacent nontumor tissues were compared using Student's *t*-test. The associations between staining index and clinicopathological features were compared using the Pearson Chi-square (*χ* ^2^) test and Fisher's exact test for categorical variables. The difference of serum AREG level between the controls and patients was compared using a Mann-Whitney *U* test. Receiver operating characteristic (ROC) curves and the area under the curve (AUC) were analyzed by *χ* ^2^ test. Survival curves were estimated by the Kalplan-Meier method. Cox's proportional hazards regression analysis was done to estimate which factors might have a significant influence on survival. *P* < 0.05 was considered to be statistically significant.

## 3. Results

### 3.1. Expression of YAP, TAZ, and AREG in HCC Tissues

**Figure 1:**
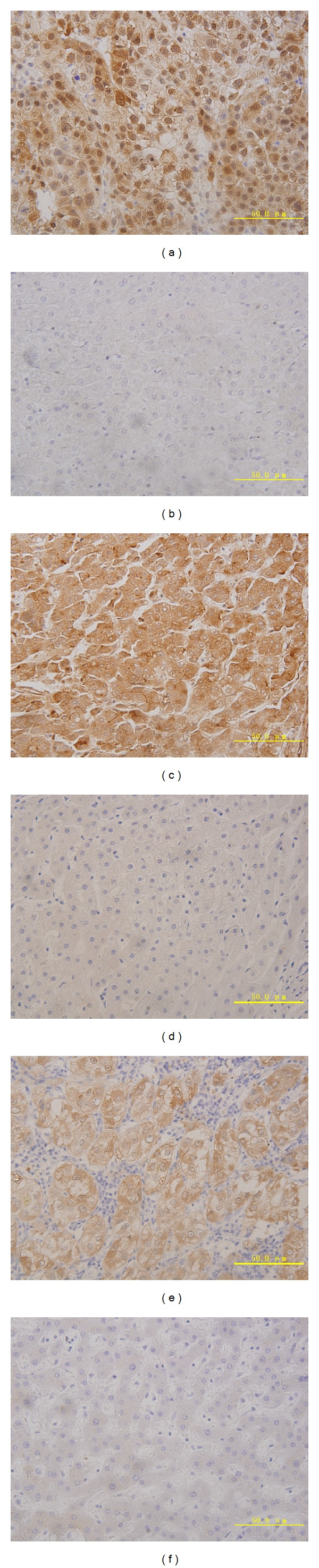
Immunohistochemically stained tissues from HCC patients and adjacent nontumor tissues. Expression of YAP in HCC tissues (a) and adjacent nontumor tissues (b); expression of TAZ in HCC tissues (c) and adjacent nontumor tissues (d); expression of AREG in HCC tissues (e) and adjacent nontumor tissues (f). Representative images were taken under a microscope (×400). These results indicate the clinical significance of YAP, TAZ, and AREG overexpression in HCC tissues.

To determine the prevalence and clinical significance of YAP, TAZ, and AREG in HCC development, we investigated their expression in 39 cases of HCC tissues and their paired adjacent nontumor tissues by immunohistochemistry. Among these patients, 69.2% (27/39) HCC tissues were positive for YAP expression in both the nuclei and cytoplasm (Figure 1(a)), whereas 41.0% (16/39) adjacent nontumor tissues were positive for YAP expression (Figure 1(b)); 66.7% (26/39) and 61.5% (24/39) of tumor tissues were, respectively, positive for TAZ and AREG (Figures 1(c) and 1(e)). In contrast, only 35.9% (14/39) and 33.3% (13/39) of these adjacent nontumor tissues were positive for TAZ and AREG expression (Figures 1(d) and 1(f)). The expression of YAP was not significantly correlated with TAZ and AREG in HCC tissues, as assessed by Spearman's rank correlation coefficient test (*P* > 0.05). There was no significant correlation between the expression of TAZ and AREG in HCC tissues (*P* > 0.05).

### 3.2. Association of YAP, TAZ, and AREG Expression with Clinico-Pathologic Characteristics of HCC Patients

The correlation between YAP, TAZ, and AREG expression in HCC and the clinicopathologic characteristics was statistically analyzed by Fisher's exact test. Expressions of YAP (Table 1) and AREG (Table 2) were significantly associated with Edmonson-Steiner classification (*P* = 0.034 and *P* = 0.001, resp.) and serum AFP level (*P* = 0.018 and *P* = 0.001, resp.). However, there were no correlations to gender, age, tumor size, portal venous invasion, number of tumor nodules, TNM stage, and HBV or HCV infection. Furthermore, no significant difference was observed between TAZ expression and these clinicopathologic features.

### 3.3. Expression of YAP in HCC Patients Correlates with Overall Survival

**Figure 2:**
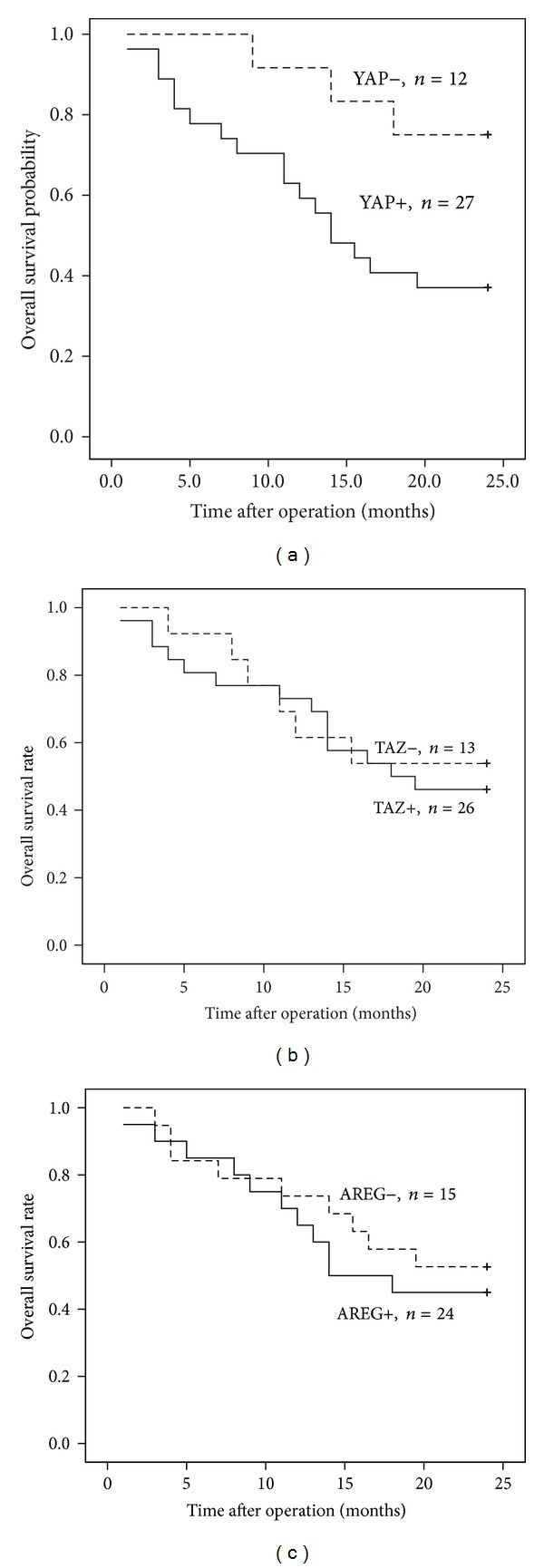
Kaplan-Meier analysis of overall survival (cumulative survival) of HCC patients relative to YAP, TAZ, and AREG expression. (a) Correlation of YAP expression with overall survival of HCC patients; (b) correlation of TAZ expression with overall survival of HCC patients; (c) correlation of AREG expression with overall survival of HCC patients.

To determine the prognostic significance of YAP and TAZ expression, we followed 39 HCC patients for two years. The overall median survival time among these patients was 19.5 months. At the end of the follow-up period 20 patients were deceased. The association between YAP and TAZ expression and overall survival of HCC patients was investigated by Kaplan-Meier analysis and log-rank test. A statistically significant difference was found between the overall survival and YAP protein expression (log-rank = 4.819, *P* = 0.028, as shown in Figure 2(a)). Univariate Cox proportional hazard analysis of protein expression and the relationship to overall survival are shown in Table 3. HCC patients with high expressions of YAP tended to have higher risk of death (*P* = 0.042). In addition, cell differentiation (Edmonson grade) and number of tumor nodules were found to be associated with HCC prognosis (as shown in Table 3). Multivariate analysis showed that higher expression of YAP was associated with reduced overall survival, adjusted HR 0.054 (*P* = 0.005). This indicates that the expression of YAP could be a prognostic factor independent of these adjusted clinicopathologic characteristics. On the other hand, the expression of TAZ (*P* = 0.722, Figure 2(b)) or AREG (*P* = 0.583, Figure 2(c)) did not predict overall survival of HCC patients.

### 3.4. Elevated Expression of Serum AREG Levels in HCC Patients and Its Significance on Diagnosis, Clinicopathology Features, and Prognosis

**Figure 3:**
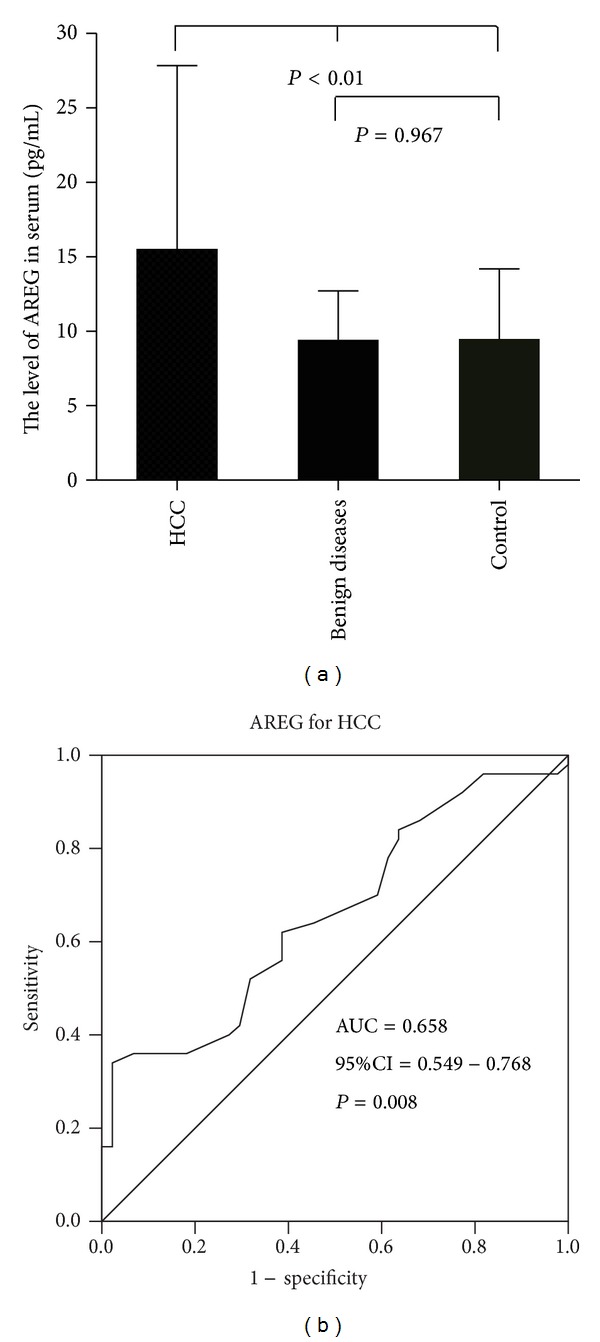
(a) showed the levels of serum AREG among normal controls, benign liver disease, and HCC patients. Mean levels of serum AREG and Std. Deviation is illustrated by bar charts; (b) showed the ROC curves of serum AREG between normal controls and HCC patients.

The expressions of serum AREG in 98 patients were determined by ELISA assay. The characteristics of 98 patients are summarized as shown in Table 4. We found serum AREG levels in HCC patients to be significantly higher than that of patients with benign liver disease and normal controls (*P* < 0.01). There was no significant difference between serum AREG levels of patients with benign liver disease and normal controls (*P* > 0.05, Figure 3(a)). Patients with HCC stages I-II did not have an elevated AREG compared to patients with HCC stages III-IV (*P* = 0.266).

ROC curves were constructed between HCC, benign liver disease, and normal controls. The AUC of AREG as diagnostic biomarkers for HCC was 0.658 (*P* = 0.008) (as shown in Figure 3(b)). A level of 14.993 pg/mL (both the sensitivity and specificity were the highest) was determined to be the most efficient threshold and was set as the cut-off value. The sensitivity of the assay was 34% and the specificity was 97.7%. The positive and negative predictive values were 94.4% and 58.7%, respectively. The specificity of serum AREG in the diagnosis of HCC was higher than that of AFP.

**Figure 4:**
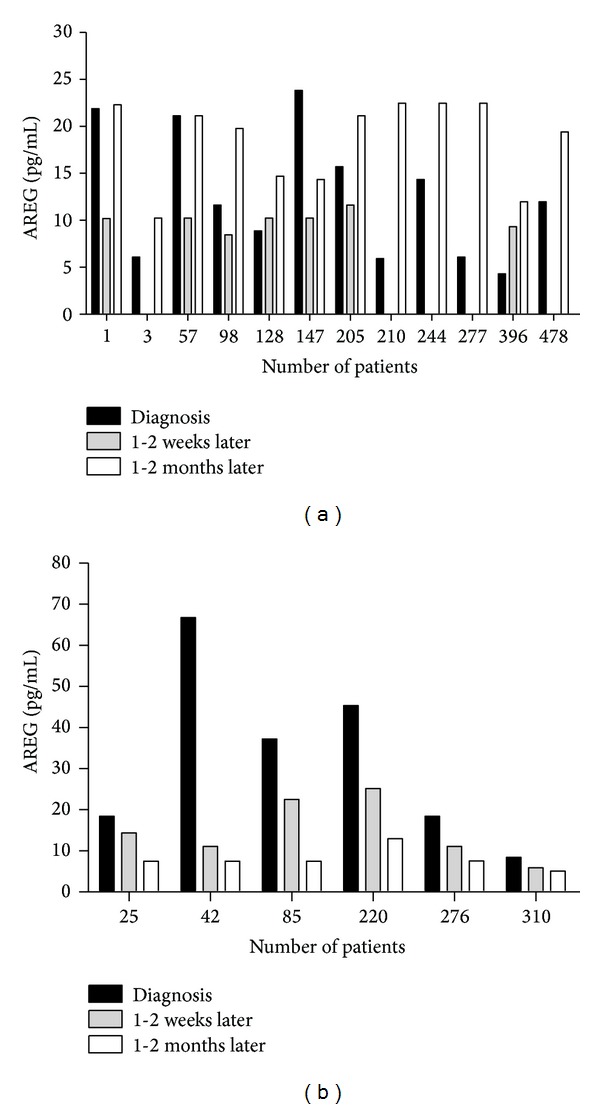
(a) Twelve patients with HCC who underwent postoperative adjunctive therapy during two-year follow-up. At last all of them had tumor recurrence or metastasis, and some of them were dead. The sample of postoperation 1-2 weeks of patients number 3/210/277 was not collected; (b) levels of sera AREG from another six HCC patients were showed after operation and during follow-up.

Next, we examined AREG expression in HCC tissues (as shown in Figures 1(e) and 1(f)). 61.5% (24/39) of HCC tissues had high AREG expression and 56.0% (28/50) of HCC serum from patients had high serum AREG levels above the cut-off value of 14.993 pg/mL. In order to determine whether concentration of serum AREG would change with the development of clinical treatment, a follow-up study was performed over a two-year period. In the study, 47 of 50 HCC patients were observed over the course of two years (3 HCC patients lost to follow-up) and sera were collected from these 47 HCC patients in 1-2 weeks after tumor resection, postoperative chemotherapy, or hepatic arterial chemotherapy. During the follow-up of 47 HCC patients, 18 HCC patients underwent operation or transcatheter arterial chemoembolization (TACE) treatment. Of these 18 HCC patients, serum AREG levels were elevated in 12 HCC patients before operation and decreased in 5 HCC patients (5/12, 41.7%) after operation, while being slightly elevated in 2 HCC patients and significantly elevated in 9 patients after TACE. At last, 12 HCC patients were observed with tumor recurrence and metastasis (as shown in Figure 4(a)) (We are not able to get the detailed clinical information. This is a limitation of this study.) In another six HCC patients, serum AREG level decreased after operation and TACE (as shown in Figure 4(b)). They had better prognosis compare to above 12 HCC patients. Of 47 HCC patients, 20 HCC patients were deceased in the end of follow-up.

### 3.5. Expression of YAP in HCC Is an Independent Prognostic Factor for Poor Survival Outcome

Multivariate analysis of 39 HCC patients performed different variables (Table 3), including serum AFP levels, Edmondson stage, YAP expression, TAZ expression, AREG expression, and the level of serum AREG. Multivariate analysis results showed that only YAP expression was an independent prognostic factor significantly associated with overall survival of HCC patients. HCC patients with positive YAP expression had higher risk (*P* = 0.005) for overall survival compared to those with negative YAP expression. However, the expressions of TAZ, AREG, serum AREG were not independent prognostic factors in these HCC patients.

## 4. Discussion 

The role of controlling organ size in HCC development has attracted recently attention. The Hippo signaling pathway has been shown to be critical in controlling organ size in mammals. The Hippo kinase cascade, a signaling pathway that antagonizes YAP, plays an important role in animal organ size controlling by regulating cell proliferation and apoptosis rates. During the development of HCC, the pathway is likely inactivated in tumor initiated cells that escape suppressive constrain exerted by the surrounding normal tissue, thus allowing clonal expansion and tumor development [6]. YAP, TAZ, and AREG are downstream effectors of the Hippo pathway and have been reported either as oncogene candidates or tumor suppressors. However the role of these proteins in cancer is not clearly known.

Hepatocarcinogenesis is a long-term, complex process involving multiple risk factors and different genetic alterations that occur during initiation, promotion, and progression of the disease [11]. The role of hepatitis B virus (HBV) infection in developing HCC is well accepted, and HBV X protein (HBx) plays critical roles in the development of HCC. Recent study showed that YAP expression was dramatically elevated in clinical HCC samples, HBV infected hepatic cell line, and liver cancer tissues of HBx transgenic mice. Hepatitis C virus (HCV) infection is also associated with the development of HCC [12]. However, the results also showed that HBV or HCV infection did not affect YAP expression in HCC. Overexpression of YAP induced epithelial-to-mesenchymal transition (EMT) and suppressed apoptosis, growth factor-independent proliferation, and anchorage-independent growth [13]. Our study also showed that YAP is overexpressed in HCC and correlated with higher Edmondson stage and higher serum AFP level. Collectively, these results suggest that YAP can be considered as an independent prognostic marker of HCC. In fact, YAP has been implicated as an oncogene and is altered in different kinds of human digestive system cancers, especially hepatocellular carcinoma. Zhao et al. found that 54% (63/115) of HCC tissues showed strong YAP staining, while 95% of normal liver tissues showed very weak YAP staining. Further study in a HCC cohort demonstrated that both YAP protein and mRNA levels were significantly increased in the majority of HCC tissue compared with adjacent nontumor tissue [14–16]. These results and our results suggest that YAP plays an important role in human HCC. Moreover, the idea that the overexpression of YAP leads to HCC development was confirmed by experiments in YAP transgenic mice [17].

TAZ, as the paralog of YAP, has similar functions to YAP. TAZ increases the expression of several genes that promotes mesenchymal differentiation in malignant glioma [18]. As a result, the cells lose their epithelial properties including presence of polarity, intercellular junctions, and acquire mesenchymal or stem cell-like properties. TAZ also plays an important role in migration, invasion, and tumorigenesis of breast cancer cells and is overexpressed in about 20% of human breast cancers [4]. TAZ also contributes to the oncogenesis of nonsmall cell lung cancer [19]. Tumors with high expression of TAZ are more invasive and metastatic and therefore difficult to treat. Our results for the first time demonstrated that there is no relationship between TAZ expression in HCC and the clinicopathologic features described.

AREG is expressed in human hepatocellular carcinoma tissues and cell lines and behaves as a mitogenic and antiapoptotic growth factor for hepatocarcinoma cells. Increasing evidence has shown that AREG activated the EGFR which is an important mechanism in the development of hepatocarcinoma. Recent evidences suggest that AREG can play a unique role in liver tumorigenesis and in the maintenance of the neoplastic phenotype of hepatocarcinoma cells [20] and thus constitutes a novel therapeutic target in human hepatocarcinoma [21]. Our results also demonstrated the same conclusion. In this study, elevated expression of AREG in HCC was demonstrated and was related to the lower Edmondson stage and lower circulation AFP. In addition, the level of serum AREG was upregulated and acted as a biomarker for early diagnosis, but there was no correlation between serum AREG level and tissue AREG expression levels in HCC.

Taken together, in this investigation, we examined the expression of TAZ, YAP, and AREG in HCC and its clinical prognostic significance. Although the patient population was limited and we are not able to get the detailed clinical information of HCC patients, our findings are of clinical significant because it opens a novel area of research for further investigation in HCC.

## 5. Conclusion

YAP was an independent prognostic indicator for HCC patients. Serum AREG was a serological biomarker in the diagnosis of HCC. Further investigation is desirable for dissecting the complicated disease of HCC.

## Figures and Tables

**Table 1:** Clinical correlation of YAP expression in HCC.

Clinicopathologic features	Total number of patients, *N*=	YAP expression		*χ* ^2^	*P*
		−	+		
Age (mean ± SD), years	39 (51.77 ± 1.93)				
<55	23	6	17	0577	0.498
≥55	16	6	10		
Sex					
Men	35	10	25	0.774	0.573
Women	4	2	2		
Tumor size, cm					
>5	20	5	15	0.641	0.501
≤5	19	7	12		
Edmonson grade					
I+II	26	11	15	4.875	0.034*
III	13	1	12		
Venous infiltration					
Present	6	3	3	1.231	0.348
Absent	33	9	24		
Hepatitis					
Negative	9	5	4	3.374	0.102
HBV/HCV	30	7	23		
Serum AFP level, ng/mL					
≤200	21	10	11	6.064	0.018*
>200	18	2	16		
TNM stage					
I + II	15	5	10	0.075	1.000
III + IV	24	7	17		
Number of tumor nodules					
1	36	10	26	1.966	0.219
≥2	3	2	1		

YAP indicates yes-associated protein; HCC: hepatocellular carcinoma; SD: standard deviation; HBV: hepatitis B virus; HCV: hepatitis C virus; AFP: *α*-fetoprotein; TNM: tumor node metastasis.

Tumor size was measured by the length of the largest tumor nodule.

*Statistically significant.

**Table 2:** Clinical correlation of AREG expression in HCC.

Clinicopathologic features	Total number of patients, *N*=	AREG expression		*χ* ^2^	*P*
		−	+		
Age (mean ± SD), years	39 (51.77 ± 1.93)				
<55	23	8	15	0.321	0.740
≥55	16	7	9		
Sex					
Men	35	13	22	0.251	0.631
Women	4	2	2		
Tumor size, cm					
>5	20	9	11	0.742	0.514
≤5	19	6	13		
Edmonson grade					
I + II	26	5	21	12.188	0.001*
III	13	10	3		
Venous infiltration					
Present	6	3	3	0.399	0.658
Absent	33	12	21		
Hepatitis					
Negative	9	4	5	0.177	0.711
HBV/HCV	30	11	19		
Serum AFP level, ng/mL					
≤200	21	3	18	11.236	0.001*
>200	18	12	6		
TNM stage					
I + II	15	6	9	0.024	1.000
III + IV	24	9	15		
Number of tumor nodules					
1	36	13	23	1.092	0.547
≥2	3	2	1		

AREG indicates amphiregulin protein; HCC: hepatocellular carcinoma; SD: standard deviation; HBV: hepatitis B virus; HCV: hepatitis C virus; AFP: *α*-fetoprotein; TNM: tumor node metastasis.

Tumor size was measured by the length of the largest tumor nodule.

*Statistically significant.

**Table 3:** Cox regression analysis of overall survival (*n* = 39).

	*N* =	Univariate analysis			Multivariate analysis		
		HR	95% CI	*P*	HR	95% CI	*P*
YAP							
Negative	12	1			1		
Positive	27	0.279	(0.081 − 0.955)	0.042*	0.054	(0.007 − 0.413)	0.005*
TAZ							
Negative	13	1			1		
Positive	26	0.842	(0.323 − 2.194)	0.725	1.962	(0.514 − 7.499)	0.324
AREG							
Negative	15	1			1		
Positive	24	0.783	(0.324 − 1.893)	0.588	0.436	(0.144 − 1.321)	0.142
Age							
<55	23	1			1		
≥55	16	0.636	(0.264 − 1.532)	0.313	0.629	(0.182 − 2.176)	0.464
Sex							
Men	35	1			1		
Women	4	2.661	(0.356 − 19.894)	0.340	3.158	(0.330 − 30.219)	0.318
Tumor size, cm							
≤5	19	1			1		
>5	20	0.524	(0.208 − 1.317)	0.169	2.049	(0.325 − 12.896)	0.445
Edmonson grade							
I + II	26	1			1		
III	13	0.403	(0.167 − 0.975)	0.044*	0.846	(0.200 − 3.680)	0.820
Venous infiltration							
Present	6	1			1		
Absent	33	0.559	(0.186 − 1.680)	0.300	0.187	(0.025 − 1.422)	0.105
Hepatitis							
Negative	9	1			1		
HBV/HCV	30	1.692	(0.649 − 4.412)	0.282	8.851	(1.667 − 47.008)	0.010*
Serum AFP level, ng/mL							
≤200	21	1			1		
>200	18	0.896	(0.373 − 2.154)	0.896	1.141	(0.384 − 3.387)	0.812
TNM stage							
I + II	15	1			1		
III + IV	24	0.427	(0.155 − 1.179)	0.101	0.234	(0.036 − 1.511)	0.127
No. of tumor nodules							
1	36	1			1		
≥2	3	0.242	(0.069 − 0.844)	0.026*	0.143	(0.027 − 0.752)	0.022*

95% CI indicates 95% confidence interval.

*Statistically significant.

**Table 4:** Characteristics of 98 patients for serum AREG.

	*n*	Range of the age (years)	Mean of the age (years)
Normal controls	17	33–67	53.00
Benign liver diseases	31	26–69	50.23
Clinical stages	50	13–74	51.52
HCC I + II	20	26–63	49.30
HCC III + IV	30	13–74	52.55

Total	98	13–74	50.22

Stratifying the analysis by American Joint Committee on Cancer stage for HCC.
